# Solid Lipid Nanoparticles Containing Morin: Preparation, Characterization, and Ex Vivo Permeation Studies

**DOI:** 10.3390/pharmaceutics15061605

**Published:** 2023-05-28

**Authors:** Federica De Gaetano, Consuelo Celesti, Giuseppe Paladini, Valentina Venuti, Maria Chiara Cristiano, Donatella Paolino, Daniela Iannazzo, Vincenza Strano, Anna M. Gueli, Silvana Tommasini, Cinzia Anna Ventura, Rosanna Stancanelli

**Affiliations:** 1 Department of Chemical, Biological, Pharmaceutical and Environmental Sciences, University of Messina, V.le Ferdinando Stagno d’Alcontres 31, 98166 Messina, Italy; fedegaetano@unime.it (F.D.G.); stommasini@unime.it (S.T.); rstancanelli@unime.it (R.S.); 2 Department of Engineering, University of Messina, Contrada Di Dio, 98166 Messina, Italy; ccelesti@unime.it (C.C.); daniela.iannazzo@unime.it (D.I.); 3 Department of Physics and Astronomy “Ettore Majorana”, University of Catania, Via S. Sofia 64, 95123 Catania, Italy; giuseppe.paladini@unict.it (G.P.); anna.gueli@unict.it (A.M.G.); 4 Department of Mathematical and Computer Sciences, Physical Sciences and Earth Sciences, University of Messina, V.le Ferdinando Stagno D’Alcontres 31, 98166 Messina, Italy; vvenuti@unime.it; 5 Department of Medical and Surgical Sciences, University of Catanzaro “Magna Graecia”, V.le Europa s.n.c., 88100 Catanzaro, Italy; mchiara.cristiano@unicz.it; 6 Department of Experimental and Clinical Medicine, University of Catanzaro “Magna Graecia”, V.le Europa s.n.c., 88100 Catanzaro, Italy; paolino@unicz.it; 7 National Council of Research, Institute of Microelectronics and Microsystems (CNR-IMM), University of Catania, Via S. Sofia 64, 95123 Catania, Italy; vincenzina.strano@ct.infn.it

**Keywords:** morin, solid lipid nanoparticles, in vitro release, physical–chemical characterization, ex vivo permeation

## Abstract

In recent years, bioactive compounds have been the focus of much interest in scientific research, due to their low toxicity and extraordinary properties. However, they possess poor solubility, low chemical stability, and unsustainable bioavailability. New drug delivery systems, and among them solid lipid nanoparticles (SLNs), could minimize these drawbacks. In this work, morin (MRN)-loaded SLNs (MRN-SLNs) were prepared using a solvent emulsification/diffusion method, using two different lipids, Compritol^®^ 888 ATO (COM) or Phospholipon^®^ 80H (PHO). SLNs were investigated for their physical–chemical, morphological, and technological (encapsulation parameters and in vitro release) properties. We obtained spherical and non-aggregated nanoparticles with hydrodynamic radii ranging from 60 to 70 nm and negative zeta potentials (about −30 mV and −22 mV for MRN-SLNs-COM and MRN-SLNs-PHO, respectively). The interaction of MRN with the lipids was demonstrated via μ-Raman spectroscopy, X-ray diffraction, and DSC analysis. High encapsulation efficiency was obtained for all formulations (about 99%, *w*/*w*), particularly for the SLNs prepared starting from a 10% (*w*/*w*) theoretical MRN amount. In vitro release studies showed that about 60% of MRN was released within 24 h and there was a subsequent sustained release within 10 days. Finally, ex vivo permeation studies with excised bovine nasal mucosa demonstrated the ability of SLNs to act as a penetration enhancer for MRN due to the intimate contact and interaction of the carrier with the mucosa.

## 1. Introduction

Neurodegenerative diseases such as Alzheimer’s, Parkinson’s, Huntington’s, multiple sclerosis, and ischemic stroke represent a threat to world health, and the incidence of these pathologies dramatically increases with age [1,2]. These disorders commonly lead to the irreversible destruction of neuronal networks, resulting in permanent damage to the central nervous system [3]. It has been shown that these pathologies are related to the formation of reactive oxygen species (ROS) and/or reactive nitric species (RNS) [4]. Their presence can damage cellular components and cause a state of oxidative stress [5,6,7].

Antioxidants are substances acknowledged as “free radical scavengers”, able to reduce free radicals and reduce the onset of aging, cancer, diabetes, inflammation, liver disease, cardiovascular disease, cataract, nephrotoxicity, and neurodegenerative disorders [8,9]. Several in vitro and in vivo research studies have highlighted the potential of natural antioxidants [10,11,12], because these products can contribute to facing oxidative stress [13], eliminate abnormal protein aggregates [14], and have effects of neurite outgrowth, memory improvement, neuroprotective, axonal regeneration, and synaptic reconstruction [3].

Preclinical studies have shown positive effects of flavonoids on these pathologies and have suggested their use in therapy [15]. Flavonoids are a large group of naturally occurring polyphenolic compounds [16], whose antioxidant and free radical scavenging activities are determined by the position on the structure of the hydroxy, methoxy, geranyl, or other group substitutions [17]. As demonstrated by Zima et al. [18], numerous flavanones isolated from *Paulownia tomentosa* show in vitro antiradical activity and in vivo cytoprotective effects against alloxan-induced diabetes. Morin (MRN, 3,5,7,2′,4′-pentahydroxyflavone), derived from the Moraceae family, is a flavonoid which is biologically active in various human pathologies. MRN exerts strong anticancer effects against different types of cancers [19], such as cervical, breast, colon, prostate, liver, and melanoma. Recently, it has also been shown to exert antioxidant [20], anti-inflammatory [21], anti-aggregation (toward Aβ fibrils) [22], and neuroprotective effects [23,24], thus improving behavioral outcome, histological structure, and neuronal survival in adult rats suffering from brain trauma [25]. Furthermore, MRN could be useful for the treatment of neurological disorders of vascular or degenerative origin, or as an adjuvant to enhance the action of other cerebrotonic drugs. Despite these beneficial effects, MRN has low water solubility (28 μg/mL) [26] and high first-pass metabolism, which reduce its oral bioavailability [27], thus limiting its clinical application. Recently, as an alternative to oral administration, intranasal administration received great interest, representing a non-invasive route of administration that can permit the delivery of drugs directly to the brain, by means of the olfactory and trigeminal nerves’ pathway [28,29]. However, drugs administered via the intranasal route must have adequate water solubility to achieve a high concentration in the mucus layer present in the nasal mucosa. Furthermore, the small volume of the nasal cavity and the rapid muco-ciliary clearance, which significantly reduce the residence-time of the drug applied on the nasal mucosa, make nose-to-brain drug delivery challenging [30]. In this context, the use of supramolecular drug delivery systems, such as cyclodextrins [31,32], polymeric [33,34], or lipidic nanoparticles [35], can be helpful in overcoming these problems related to the unfavorable physical–chemical properties of the drug and its rapid elimination from the nasal cavity [36]. Among them, solid lipid nanoparticles (SLNs), due to their low toxicity and biodegradability, have been reported to be suitable systems for various application routes [37]. Due to their lipophilic nature and very low sizes, they can easily squeeze through intercellular spaces between olfactory cells [38], rapidly reaching the central nervous system [39], showing high efficacy as carriers for the nose-to-brain targeting of different drugs. Pharmacokinetic studies on albino Wistar rats demonstrated that the intranasal administration of haloperidol-loaded SLNs produced a higher maximum concentration in the brain, with respect to the free drug administered via the same route or intravenously [40]. In addition, SLNs have been reported [37,41,42,43] to protect different types of drugs from biological and/or chemical degradation, exhibiting at the same time a rapid and direct nasal–brain transfer, bypassing the blood–brain barrier.

Different flavonols [44,45] and, amongst them MRN have also recently been encapsulated in SLNs with the aim of enhancing their biological properties, such as their anticancer activity [46], or even increasing their permeation via the intestinal membrane [47].

On these bases, a preformulation study was performed to realize inhalable dry-powder MRN-loaded SLNs (MRN-SLNs) able to increase the nasal permeation of the drug, and above all to obtain a prolonged release of MRN in the brain. MRN-SLNs were obtained via a solvent emulsification/diffusion method using Compritol^®^ 888 ATO (COM) or Phospholipon^®^ 80H (PHO) as the lipid and Polysorbate 80 as the surfactant. A deep characterization study was carried out in order to achieve the best formulation in terms of sizes, electric surface properties, encapsulation efficiency, drug loading, and release rate. Scanning electron microscopy (SEM) was used for morphological characterization. A detailed physical–chemical characterization of the systems was conducted via μ-Raman spectroscopy, differential scanning calorimetry (DSC), and thermogravimetric (TG) analyses. Finally, ex vivo permeation studies of excised bovine nasal mucosa were carried out to evaluate the ability of the delivery systems to improve and prolong the permeation of MRN through the mucosa.

## 2. Materials and Methods

### 2.1. Materials

Compritol^®^ 888 ATO (COM) was kindly furnished by Gattefossé SAS (Lyon, France); Phospholipon^®^ 80H (PHO) was purchased from Phospholipid GmbH (Cologne, Germany). Morin (MW 302.24), Tween^®^ 80, and trehalose with Saccharomyces Cerevisiae ≥99% were purchased from Sigma Aldrich (St. Louis, MO, USA). Methanol, ethanol, and acetonitrile HPLC grade were Merck^®^ (Darmstadt, Germany) products. Acetone HPLC grade was purchased from J.T. Baker^®^ (Radnor, PA, USA). Double-distilled water filtered through 0.22 μm Millipore^®^ GSWP filters (Bedford, MA, USA) was used throughout the study. All other products and reagents were of analytical grade. Dialysis bags were Spectra/Por^®^ 18 mm × 11.5 mm Dialyse-Membrane (MWCO: 1000, Spectrum Laboratories, Inc., Rancho Dominguez, CA, USA).

### 2.2. Preparation of Blank SLNs and MRN-SLNs

The preparation of the blank SLNs was performed according to the solvent emulsification/diffusion method reported by Butani et al. [48], with slight modification.

Briefly, two phases were prepared: (i) a lipid phase, formed of 50 mg of PHO or 50 mg of COM solubilized in 2.5 mL methanol, (ii) an aqueous phase, formed of 11.25 mL of water containing 170 μL of Tween^®^ 80 (0.5% *w*/*v*) [49]. Both solutions were heated in a water bath; in particular, the COM solution was heated at a temperature above 80 °C and the PHO solution was heated at a temperature above 60 °C. The lipid phase was added dropwise during the aqueous phase, during high-speed homogenization, using Ultra-Turrax T 25 (IKA-Werke, Staufen, Germany) at 11,000 rpm for 7 min. To reduce the sizes of the samples, the suspensions underwent ultrasonication (Bandelin Sonorex^®^ RK 514, amplitude delivered ranges between 3 and 5 μm, Berlin, Germany) for 5 min. After that, they were allowed to cool to room temperature under magnetic stirring (500 rpm) for 24 h to permit the evaporation of the organic solvent. Purification of the samples was carried out via centrifugation at 5000 rpm for 30 min (Heraeus Megafuge 16, ThermoFisher scientific, Waltham, MA, USA) to remove the lipids that did not interact. The obtained pellets were discarded, while the supernatant, containing the SLNs, was collected; 5% (*w*/*v*) trehalose as a cryoprotectant was added, and it was freeze-dried for 72 h (BenchTop K Series Freeze Dryers, VirTis, Gardiner, NY, USA).

MRN-SLNs were prepared according to the aforementioned procedure, with the addition of different concentrations of MRN to the lipid phase (2%, 5%, 8%, and 10%, *w/w*, based on lipid). The pellets obtained from the centrifugation at 5000 rpm for 30 min, and containing the lipids that did not interact and the drug that was not encapsulated, were collected and used for the indirect determination of MRN encapsulated in SLNs. Trehalose (5%, *w*/*v*) was added to the supernatant and the MRN-SLNs were lyophilized for 72 h.

### 2.3. Technological Characterization of MRN-SLNs

Freeze-dried blank SLNs and MRN-SLNs based on COM (MRN-SLNs-COM) or on PHO (MRN-SLNs-PHO) were weighed, and the yield percentage was determined following the formula in Equation (1):

Yield (%) = (Effective yield/Theoretical yield) × 100(1)

The encapsulation efficiency (E.E.) and drug content (D.C.) percentage of the MRN-SLNs were indirectly determined via the quantification of the drug in the pellets; then, the following equations, Equations (2) and (3), were applied:

E.E. (%) = [(MRN initially addedm − free MRN outside SLNs)/ MRN initially added] × 100(2)

D.C. (%) = [(MRN initially added−free MRN outside SLNs)/ recovered SLNs] × 100(3)

To determine the amount of free MRN, the pellets derived from the centrifugation at 5000 rpm were treated with cold methanol, since this organic solvent permits the solubilization of the drug, leaving the lipid insoluble. Hence, this suspension was filtered and analyzed via HPLC (Section 2.11).

The results are reported as the mean of three separate measurements on three different batches ± the standard deviation (S.D.).

The hydrodynamics radius, (R_H_), polydispersity index (PDI), and zeta potential (ζ) of the MRN-SLNs-COM and MRN-SLNs-PHO were determined via dynamic light scattering measurements analyses, using the Zeta sizer 3000 instrument (Malvern Panalytical Ltd., Malvern, UK).

### 2.4. Thermogravimetric Analysis

Thermogravimetric analysis (TGA) was performed using the TAQ500 instrument (TA Instruments, New Castle, DE, USA) under argon flow at a flow rate of 100 mL/min. All samples tested, after a vacuum drying procedure, were heated up to 700 °C with a heating rate of 10 °C/min.

### 2.5. Differential Scanning Calorimetry Analysis

Differential scanning calorimetry (DSC) was performed using the TAQ500 instrument (TA Instruments, New Castle, DE, USA) under nitrogen flow at a flow rate of 50 mL/min, from room temperature to 350 °C, with a heating rate of 5 °C/min.

### 2.6. X-ray Diffraction Analysis

All X-ray diffraction (XRD) experiments were performed at room temperature using a Bruker D8 Advance diffractometer (Bruker, Karlsruhe, Germany) and a Bragg–Brentano theta-2theta configuration, with Cu Ka radiation (40 V, 40 mA). XRD patterns were collected in the range of 5–80° with a step of 0.2°/s. Diffraction peaks were compared with those in the Joint Committee on Powdered Diffraction Standards (JCPDS) database.

### 2.7. Scanning Electron Microscopy (SEM) Analysis

The morphology of the samples was investigated using SEM analysis (Gemini field emission SEM, Carl Zeiss Supra 25). The acceleration voltage was varied in the range of 2–5 kV and the analyses were performed using Everhart–Thornley and in-lens detectors. The freeze-dried SLNs were dispersed in deionized water; then, a drop of the suspensions was cast onto a conductive substrate and dried in air. To limit the local charging induced by the electron beam, the samples were coated with a thin gold film (~10 nm) via sputtering deposition.

### 2.8. μ-Raman Spectroscopy Analysis

μ-Raman analysis was conducted on MRN, PHO, COM, MRN-SLNs-PHO, and MRN-SLNs-COM solid systems using a “BTR 111 Mini-RamTM” (B&W Tek, Inc., Newark, NJ, USA) portable spectrometer, with a diode laser source operating at 785 nm, maximum laser power at the excitation port of 280 mW, and a charge-coupled device (CCD) detector (thermoelectric cooled, TE). Spectra were recorded in the 500–1800 cm^−1^ range using a resolution of 10 cm^−1^, and an acquisition time of 10 s × 32 scans. Calibration was performed by means of the peak of a silicon chip at 520.6 cm^−1^, thus guaranteeing the best instrument performance. The system was equipped with a BAC151B Raman microscope. An 80× objective was used, with a working distance of 1.25 mm and laser beam spot size of 26 μm. The maximum power delivered to the samples was ~120 mW.

### 2.9. In Vitro Release Studies

The release of MRN from SLNs was carried out using the most versatile and popular dialysis bag method [50,51,52], already described by our research group [44]. A weighted amount of freeze-dried MRN-SLNs-PHO and MRN-SLNs-COM formulations (both containing 5 mg of MRN) was suspended in 5 mL of phosphate-buffered solution (PBS, pH 7.4) and placed into the dialysis bag, previously wetted in PBS (pH 7.4) for 24 h. Hence, the bag was immersed in 150 mL of PBS (pH 7.4) containing 15% (*v*/*v*) ethanol as the dialysis medium and left under magnetic stirring (300 rpm) at 37.0 ± 0.5 °C (Telesystem 15.40 thermostatic bath with a Telemodul 40 °C control unit). At fixed time intervals (0.5, 1, 3, 5, 24, 48, 72, 120, 168, 240 h), the dialysis medium was removed and replenished with a fresh PBS/ethanol (15%, *v*/*v*) mixture, at the same temperature, to maintain the sink conditions. The dosage of the released MRN was performed via HPLC (Section 2.11).

#### In Vitro Release Kinetics Analysis

Data obtained via in vitro release studies were analyzed using different mathematical model equations, i.e., the zero-order Equation (4), first-order Equation (5), Higuchi Equation (6), and Korsmeyer–Peppas Equation (7) [53,54].

Q_t_ = Q_0_ + k_0_t (4)

Log C = log C_0_ − k_t_/2.303 (5)

Q_t_ = k_H_ × t^1/2^ (6)

M_t_/M∞ = kt^n^ (7)

### 2.10. Ex Vivo Permeation Studies in Excised Bovine Nasal Mucosa

Ex vivo permeation studies were carried out using Franz-type diffusion cells (LGA, Berkeley, CA, USA). The bovine nasal mucosa was excised from healthy bovines (15 ± 3 months of age) immediately after slaughtering and was maintained for the appropriate time in a PBS (pH 7.4) solution containing heparin. The integrity of the nasal mucosa was monitored by measuring transepithelial electrical resistance (TEER) before and after the in vitro permeation study using a voltmeter (Millicell ERS). Each mucosa section was mounted in a Franz diffusion cell with the external face side up between the donor and the receptor compartment. The latter was filled with a hydroalcoholic acceptor solution (PBS (pH 7.4):EtOH, 60:40, *v*/*v*) to enhance the affinity of MRN for the receptor phase, guaranteeing a pseudo-sink condition (MRN solubility in the medium, at 37 ± 0.5 °C, was 4.73 mg/mL). During the entire duration of the experiments, the receptor solution was constantly stirred by a small magnetic stirring bar and maintained at 37 ± 0.5 °C. MRN (13 mM), MRN-SLNs-COM, and MRN-SLNs-PHO both containing the same amount of MRN were suspended in PBS (pH 7.4); then, 200 μL [55] of each sample was poured in the donor compartment of Franz’s cells. At prefixed times, 300 μL of the receiving solution was withdrawn and the sample volumes were replaced with the same amounts of the fresh receptor phase. All recovered aliquots were analyzed via HPLC to determine the concentration of the permeated MRN.

At the end of the permeation experiment, the amount of MRN accumulated in the mucosa was measured. The mucosa was washed with water to remove any remaining formulation, and subsequently comminuted using a surgical blade, resuspended in 5 mL of water, and homogenized at 11,000 rpm for 5 min using IKA Ultra-Turrax 1 (IKA1Werke GmbH & Co. KG, Staufen, Germany). After that, an additional minute of homogenization was performed, adding 5 mL of methanol. Each sample was centrifuged at 13,000 rpm for 30 min. The pellet was discarded, and the supernatant was collected. Then, the solvent was removed under reduced pressure, yielding residues which were added to 2 mL of methanol to solubilize the MRN. After filtration (0.22 μm Millipore filter), the solutions were injected into HPLC to determine the MRN [56,57]. The described procedure was also performed on non-treated mucosa and the final solution was injected into HPLC to determine the specificity of the method.

### 2.11. HPLC Analysis

The quantification of MRN was performed via reverse-phase HPLC (HPLC Prominence LC-20AB pump, Shimadzu North America, Columbia, MD, USA), following a method reported in the literature [58], with slight modification. Briefly, 20 μL of each sample was injected into a C-18 column (Discovery C18 column, 250 mm × 4.6 mm inner diameter (i.d.), 5 μm, Supelco) and the analysis was performed at a flow rate of 1 mL/min using a mobile phase consisting of 0.5% acetic acid in water/acetonitrile (80:20, *v*/*v*). MRN was detected at 355 nm using a UV–Vis detector (Shimadzu UV–Vis detector SPD-20A). A chromatogram of MRN is reported in the Supplementary Materials (Figure S1), as an example.

### 2.12. Analytical Method Validation

The HPLC method was validated for linearity, specificity, sensitivity, and repeatability. A stock solution of MRN was prepared by dissolving 10 mg of the drug in 100 mL of methanol in a calibrated flask. Standard solutions were prepared at concentrations ranging from 2 μg/mL to 400 μg/mL, starting from stock solution. Six replicates of each concentration were prepared and analyzed in triplicate. The calibration curve was linear in the range of 10–400 μg/mL, obtaining an R^2^ value of 0.998 (Figure S2 in Supplementary Materials). The limit of detection (LOD) and limit of quantitation (LOQ) were determined via the signal-to-noise ratio [59], obtaining 3 μg/mL (corresponding to 3:1 signal-to-noise ratio) and 10 μg/mL (corresponding to 10:1 signal-to-noise ratio) for LOD and LOQ, respectively. The precision of the method was determined intraday and interday by injecting three of the calibration standards (10, 200, 400 μg/mL) six times during the same day and for the next five days. High intraday (RSD, 0.55%) and interday (RSE, 0.45%) precision was obtained. To determine the specificity of the method, a methanol solution derived from the extraction procedure on treated mucosa or non-treated mucosa (see Section 2.10) and pure solvent were analyzed in triplicate. No interference was observed with the peak of MRN, demonstrating that the method was specific for the drug. No degradation of MRN was observed during analysis.

### 2.13. Statistical Analysis

All values are expressed as mean ± standard deviation (SD) and each analysis was performed three times. The results were analyzed via one-way and two-way analysis of variance (ANOVA) followed by a Bonferroni post hoc test for multiple comparisons. A value of *p* < 0.05 was considered to be significant.

## 3. Results and Discussion

MRN-SLNs, containing different theoretical amounts of MRN (2%, 5%, 8%, 10% *w*/*w*), were prepared via the solvent emulsification/diffusion method and ultrasonication, using Compritol^®^ 888 ATO (COM) or Phospholipon^®^ 80H (PHO) as lipids and Tween 80 as the surfactant. 

COM is a lipid consisting of mono-, di-, and triesters of behenic acid, with the diester fraction being predominant. Recently, it has received great interest as a lipid for SLN fabrication due to its low cytotoxicity, compared to other lipids, and ability to encapsulate a high amount of hydrophobic and hydrophilic molecules, producing their sustained release [60,61,62,63]. PHO is a natural emulsifier derived from soya oil. Despite this compound principally being used as a surfactant or as a phospholipid for liposome preparation [64], different papers reported PHO as being a co-lipid for SLN production [65,66]. Furthermore, in a previous study, we demonstrated the feasibility of PHO as a unique lipid constituent of SLNs, obtaining small particles able to produce the sustained release of the natural active rutin [44].

Before proceeding with the preparation of the systems, the stability of the drug in methanol (60 °C and 80 °C) and in a PBS (pH 7.4)/ethanol (15%, *v*/*v*) mixture was evaluated. In particular, the stability in methanol was investigated within 2 h, because it represents the time required to complete the preparation of the systems; instead, the stability of MRN in the release medium (PBS (pH 7.4)/ethanol (15%, *v*/*v*)) was carried out in 10 days at 37.0 ± 0.5 °C, leaving the drug in the dark and in the light. At fixed times, the solutions were analyzed via HPLC at 355 nm. As also reported by other authors, no significant degradation of MRN was detected for the experimental conditions, nor in the release medium [26].

In Table 1, the encapsulation parameters of the prepared SLNs are reported. Comparable yields, encapsulation efficiency, and drug content percentage were obtained for both of the systems with all of the MRN theoretical amounts tested. For all of the samples, good yields and optimum encapsulation efficiency were obtained. In particular, the best result was obtained using the highest MRN theoretical amount, with encapsulation efficiency (E.E. %) ≥ 99% (*w*/*w*) and drug content (D.C. %) of 4% (*w*/*w*), indicating the great affinity of the drug for both of the lipids used in this study. Other authors synthesized SLNs loaded with MRN using lipids other than COM and PHO, obtaining a lower E.E.% of MRN with respect to our systems (about 87% and 80%, *w*/*w*) [46,47].

Based on the good values obtained for the encapsulation parameters, to continue the successive studies, the samples with 10% theoretical amount of MRN were chosen (MRN-SLNs-COM_10_ and MRN-SLNs-PHO_10_). In Table 2, we report the hydrodynamic radius (R_H_), polydispersity index (P.D.I.), and zeta potential (ζ) values of both of the formulations, comparative to blank SLNs-COM and SLNs-PHO. The blank SLNs showed small particle sizes and homogeneous distribution (see the low values of P.D.I. in Table 2), particularly for the SLNs prepared using PHO. A slight increase in sizes was observed for both of the formulations in the presence of the drug; however, this did not affect the suitability of the delivery systems for the proposed therapeutic objective. All of the formulations showed high negative ζ values, useful for guaranteeing the high physical stability of the systems. 

**Figure 1:**
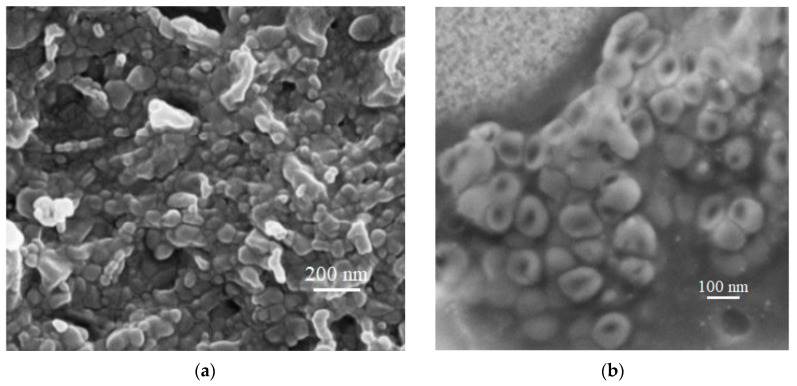
SEM images of lyophilized MRN-SLNs-COM_10_, after re-dispersion, at 200 nm (**a**) and 100 nm (**b**) magnification.

The morphology of the SLNs was investigated via SEM. In Figure 1, we show the pictures obtained for re-dispersed MRN-SLNs-COM_10_ powder. We observed spherical particles with a smooth surface and homogeneous sizes, with a very low grade of aggregation. Similar results were observed for MRN-SLNs-PHO_10_.

### 3.1. Physical–Chemical Characterization of the SLNs

The crystalline structure and polymorphic shapes of the lipids are decisive for the efficient incorporation of the drug into the SLNs and its subsequent release. These two parameters determine the effective applicability of the SLNs as drug delivery systems. Then, an exhaustive physical–chemical characterization of the systems is essential [44]. Differential scanning calorimetry (DSC), thermogravimetric analysis (TGA), and X-ray scattering are widely used to investigate the status of the lipids into SLNs.

#### 3.1.1. Differential Scanning Calorimetry

**Figure 2:**
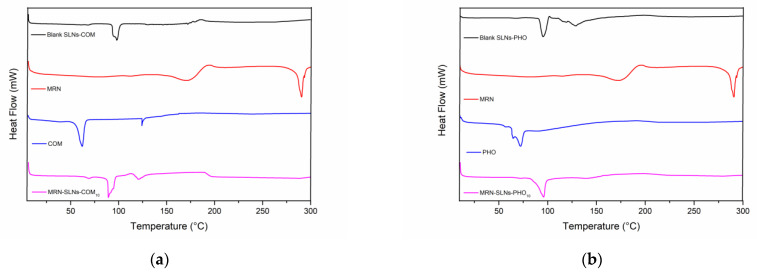
DSC thermograms of the MRN-SLNs systems: (**a**) COM, MRN, blank SLNs-COM, and MRN-SLNs-COM_10_; (**b**) PHO, MRN, blank SLNs-PHO, and MRN-SLNs-PHO_10_. The experiments were performed under an argon atmosphere.

The DSC thermograms of the investigated systems are shown in Figure 2. Both COM (Figure 2a) and PHO (Figure 2b) show three endothermic peaks, with the principal at around 60 °C and 71 °C for COM and PHO, respectively. Both lipids are a mixture of plus compounds (mono-, di-, and triglycerides of behenic acid for COM and dipalmitoyl-, distearoyl-phosphatidylcholine for PHO); so, the different endothermic peaks observed in the thermograms of COM and PHO are attributable to the melting of the different components. As a consequence of the SLNs’ formation, a shift toward a higher temperature of these peaks was observed. The thermogram of MRN shows two endothermic peaks at about 170 °C and 290 °C and an exothermal peak at about 200 °C. The latter evidences the crystallization of the drug. The first endothermic peak refers to the loss of water from the sample; the second instead refers to the melting. As observed by other authors for SLNs with different compositions [46], MRN-SLNs-COM (Figure 2a) and MRN-SLNs-PHO (Figure 2b) show the disappearance of the MRN melting peak (at about 290 °C), evidencing the interaction between the drug and the lipids, and the formation of hydrogen bonds or van der Waals interactions.

#### 3.1.2. Thermogravimetric Analysis

**Figure 3:**
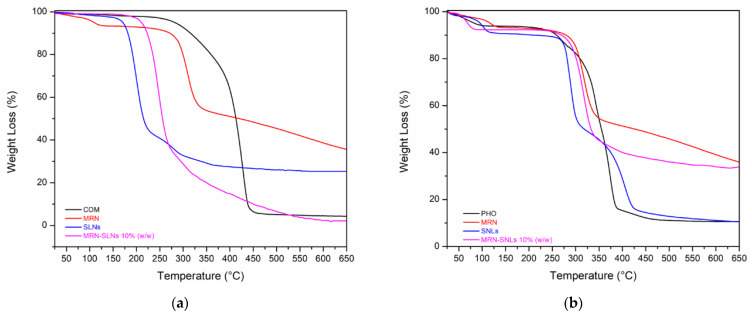
TGA thermograms of the MRN-SLNs systems: (**a**) COM, MRN, blank SLNs, and MRN-SLNs-COM_10_; (**b**) PHO, MRN, blank SLNs, and MRN-SLNs-PHO_10_. The experiments were performed under an argon atmosphere.

In this study, TGA analyses were performed in an inert environment, at the heating rate of 10 °C/min. For each sample, TGA analysis was evaluated up to 700 °C in order to investigate the thermal stability and purity of the blank SLNs and MRN-loaded SLNs. In Figure 3, we report the TGA profiles of the COM-based systems (Figure 3a) and PHO-based systems (Figure 3b).

As shown in Figure 3a, the COM sample is stable up to 350 °C; then, degradation in the range of 350–450 °C with a residual weight of 5% can be observed. The drug shows a first weight loss of about 4% in the 85–125 °C range, a second weight loss of 37% in the 250–350 °C range, and a final residual weight of 35% at the final temperature of 650 °C. The blank SNLs-COMs are stable up to 50 °C; in the 55–110 °C range, they show a first weight loss of 11%, almost certainly corresponding to the loss of absorbed water, and a second weight loss occurs in the 270–480 °C range of 56%, attributable to the SLNs-COM degradation, with a residual weight at the final temperature of 21%. The MRN-SLNs-COM_10_ sample shows an initial loss in weight in the 45–80 °C range of 10%, also attributable to absorbed water. After this first phase, it maintains a constant profile up to 200 °C; then, in the 205–550 °C range, it loses 97%, with a residual weight at the final temperature of 3%.

The graph in Figure 3b shows the weight loss profile of the MRN-SLNs-PHO_10_ systems. The PHO sample is stable up to 200 °C; after which, there is a massive weight loss in the 205–460 °C range of 90%, maintaining a residual weight at the final temperature of 10%. Three different stages of degradation are observed for the blank SLNs-PHO: the first is at 70–100 °C, attributable to water loss, the second is at 190–300 °C, with a maximum decomposition at 254 °C, and the third is at 300–400 °C, with a maximum decomposition temperature at 371 °C and a complete degradation of the sample with a total weight loss of about 90%. The MRN-SLNs-PHO_10_ sample exhibits an initial weight loss in the 50–110 °C range of 10%, attributable to absorbed water; after this first phase, it maintains a constant profile up to 250 °C. Then, in the range between 255 °C and 550 °C, it loses 65%, with a residual weight at the final temperature of 33%.

#### 3.1.3. X-ray Diffraction

**Figure 4:**
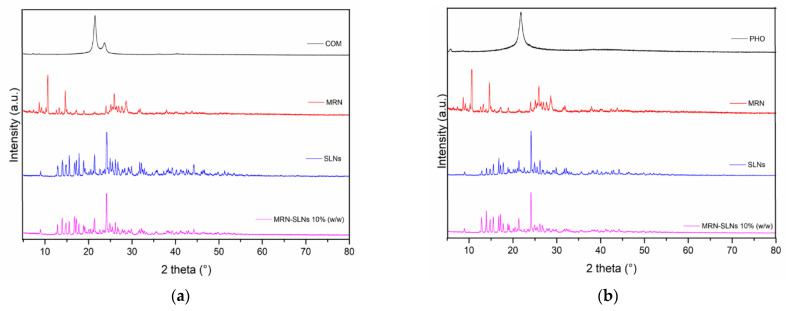
X-ray diffraction (XRD) of the MRN-SLNs systems: (**a**) COM, MRN, blank SLNs-COM, and MRN-SLNs-COM_10_; (**b**) PHO, MRN, blank SLNs-PHO, and MRN-SLNs-PHO_10_.

Both of the investigated systems show similar diffractograms (Figure 4). We observed the presence of sharp peaks at 2θ = 10.0°, 15.0°, 25.0°, and 28.0° in the diffractogram of the free drug, evidencing its crystalline status. A single diffraction peak was observed at about 2θ = 21.0° for both COM (Figure 4a) and PHO (Figure 4b). Sharp peaks were observed in the diffractograms of blank SLNs, demonstrating a different crystalline structure of the lipids in the matrix with respect to bulk solid lipids. The disappearance of the principal peaks of MRN in the diffractograms of the drug-loaded SLNs could be due to the incorporation of MRN into the crystal lattice of the lipids [67] and/or to the formation of non-covalent MRN–lipid interactions already observed via DSC analysis.

#### 3.1.4. Raman Spectroscopy

**Figure 5:**
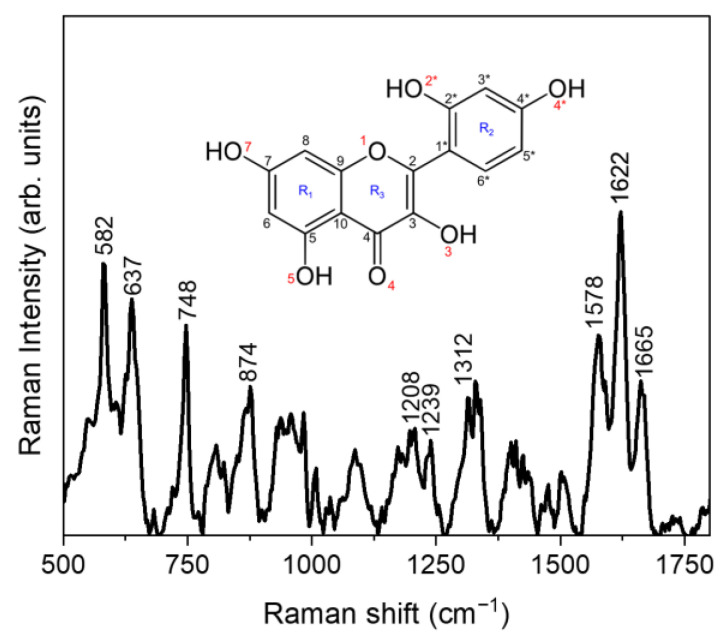
Experimental μ-Raman spectrum of MRN in the 500–1800 cm^−1^ range.

μ-Raman spectroscopy allowed us to characterize, in a solid state, the molecular interactions driving the MRN-SLNs’ formation. In particular, the used approach, already successfully applied for similar systems [68,69], consisted of monitoring the spectral changes that the vibrational bands characteristic of the free MRN undergo when the drug is loaded in the SLNs (both based on COM and PHO), as a consequence of the activation of interactions involving specific functional groups. Figure 5 shows the μ-Raman spectrum of MRN together with its molecular structure, in the range between 500 and 1800 cm^−1^, where the majority of the MRN vibrational features fall.

Based on a comparison with the literature [70], the high-intensity peaks at ~582 cm^−1^ and ~637 cm^−1^ can be ascribed to the C-C-C and C-C-O bending modes of the γ-pirone/benzene R_3_/R_2_ and benzene R_2_ rings, respectively. Furthermore, the peak at ~748 cm^−1^ results from the C3*-C4*-C5* bending mode of R_2_, while the contribution at ~874 cm^−1^ is attributable to the H3-C3*-C2* bending vibration. In addition, the contribution falling at ~1208 cm^−1^ is due to different C-O stretching vibrations of the flavonoid rings, particularly the C2*-O (in the benzene ring R_2_), C3-O (in the γ-pirone ring R_3_), and C5-O (in the benzene ring R_1_) ones. The bending vibrations of the C4*-OH and C2*-OH groups of the benzene ring R_2_ give rise, respectively, to the contributions at ~1239 cm^−1^ and ~1312 cm^−1^, while the triplet of prominent bands falling within the 1500–1750 cm^−1^ range can be assigned to the C=O (~1578 cm^−1^) stretching vibrations of the benzene ring R_2_, to the O-C (~1622 cm^−1^) stretching vibrations of the benzene and γ-pirone rings (R_1_ and R_3_), and to the C=C (~1665 cm^−1^) stretching vibrations.

**Figure 6:**
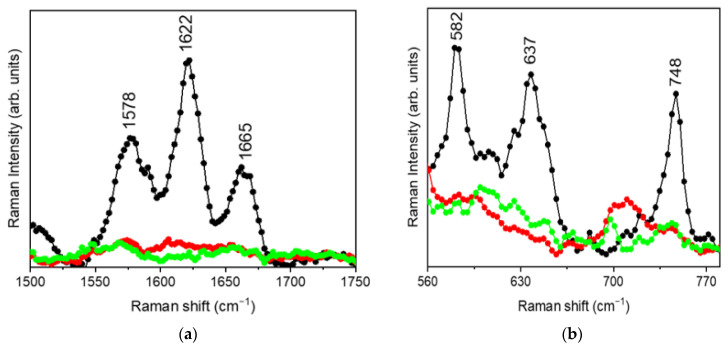
μ-Raman spectra, collected in the (**a**) 1500–1750 cm^−1^ and (**b**) 560–800 cm^−1^ range, for MRN (black dotted line), MRN-SLNs-PHO_10_ (red dotted line), and MRN-SLNs-COM_10_ (green dotted line) systems.

To obtain insights into the interactions involved in the formation of the MRN-SLNs, the regions extending from 1500 cm^−1^ to 1750 cm^−1^ and from 560 cm^−1^ to 800 cm^−1^ were focused on in detail. The choice of these spectral ranges was mainly due to the fact that they are free of vibrational contributions coming from SLNs, and hence their inspection allows one to directly monitor the variations exhibited by the vibrational bands of MRN when loaded within the SLNs. Figure 6 shows the experimental μ-Raman spectra, in the 1500–1750 cm^−1^ (a) and 560–800 cm^−1^ (b) ranges, respectively, for MRN, MRN-SLNs-PHO_10_, and MRN-SLNs-COM_10_ systems.

From the inspection of Figure 6a, a flattening of the contributions centered at ~1578 cm^−1^ (C=O stretching mode), ~1622 cm^−1^ (O-C stretching mode), and ~1665 cm^−1^ (C=C stretching mode) passing from the pristine MRN to both MRN-SLNs-PHO_10_ and MRN-SLNs-COM_10_ can be clearly observed. Such an occurrence testifies the involvement of the corresponding functional groups in the drug–nanoparticle encapsulation process, and consequently the establishment of new molecular interactions. In particular, the observed spectral modifications indicate a hindering of the aforementioned vibrational modes due the more constrained molecular environment, resulting from interactions activated during NP formation.

In addition, the analysis of the lower frequency region extending from 560 to 800 cm^−1^ (Figure 6b) also revealed a flattening of the MRN vibration bands at ~582 cm−1 (C-C-C bending mode), ~637 cm^−1^ (C-C-O bending mode), and 748 cm^−1^ (C3*-C4*-C5* bending mode) as a consequence of the drug encapsulation into SLNs. Such an occurrence testifies, in agreement with the analysis of the 1500–1750 cm^−1^ spectral region, a hindering of the aforementioned vibrational modes upon complexation, due to the establishment of new intermolecular linkages between the drug and SLNs, thus representing good evidence of complex formation.

### 3.2. In Vitro MRN Release from SLNs

**Figure 7:**
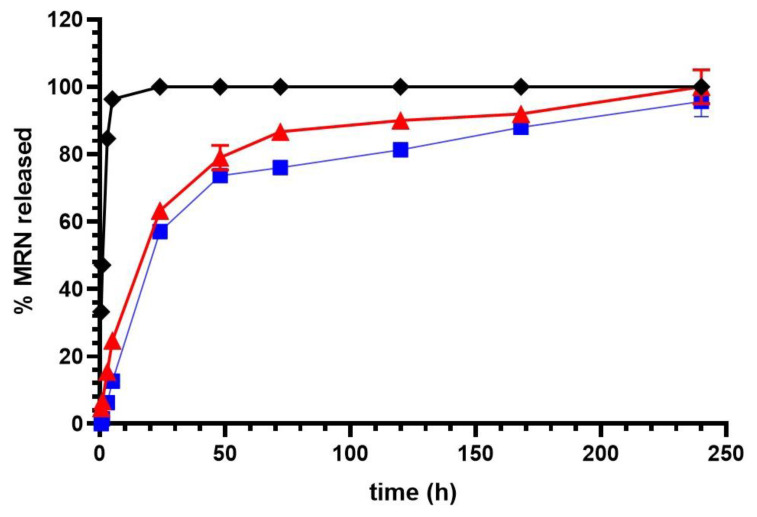
In vitro release profiles of free MRN (black line), MRN-SLNs-PHO_10_ (red line), and MRN-SLNs-COM_10_ (blue line). Experiments were carried out using the dialysis method and the results are expressed as mean values of three different experiments from three different batches ± standard deviation. If bars are not visible, they are within the symbol. All data related to MRN-SLNs are statistically significant with respect to free MRN data (*p* < 0.001).

The release of MRN from SLNs was evaluated via the dialysis method, using a mixture of PBS (pH 7.4) and ethanol (15% *v*/*v*) as the dialysis medium. Ethanol was added to ensure MRN solubilization (MRN solubility in the dialysis medium, 1.48 mg/mL). As shown in Figure 7, free MRN quantitatively crossed the synthetic membrane within 5 h, demonstrating the suitability of the chosen dialysis bag in terms of molecular weight cutoff. The fast passage of free MRN through the synthetic membrane shows that the release of the encapsulated MRN is SLN-controlled. SLNs were retained in the bag and the drugs released from the delivery systems immediately crossed the synthetic membrane. 

In Figure 7, we report the release profiles of the MRN from the two realized formulations. The most amount of encapsulated MRN was released from the SLNs within the first 24 h from the beginning of the experiment (about 18% and about 60%, *w*/*w*, was released from both of the formulations within 5 h and 24 h, respectively); the remaining amount of the drug was quantitatively released from both of the systems within 10 days. This trend could be related to the internal structure of MRN-SLNs, which presents a non-homogeneous matrix, and the shell enriched with the drug [71]. In this way, due to the limited distance, the drug present in the shell was quickly released in the medium. The prolonged subsequent release of the remaining fraction of MRN (about 40%, *w*/*w*) could be due to a homogeneous distribution within the matrix of this fraction and to the increased distance which MRN must run across to go out of the systems. Furthermore, the high affinity of the drug for the used lipids and the consequential existence of a drug–lipid association must be considered. To migrate through the lipid matrix, MRN–lipid dissociation is needed, with a consequent retarding of the diffusion process [72].

Due to the ability of SNLs to interact with the mucus and quickly be adsorbed by the nasal mucosa [73], SLNs can avoid mucociliary clearance and translate into the brain via different routes [30]. In this way, the amount of MRN released within the first few hours could assure the initial therapeutical effect of MRN when SLNs reach the brain. The subsequent sustained release could be useful to prolong the therapeutical activity of MRN and avoid frequent administrations, increasing the patient’s compliance. 

To investigate the MRN release mechanism from the SLNs, the release data were treated according to different kinetic models [74,75,76]. The best-fit model was achieved by comparing the regression coefficient (R^2^) value of all of the models; in general, the closer the R^2^ value was to 1, the better fit or relationship between the two factors (Table 3). The first-order kinetic model shows higher R^2^ values than the zero-order kinetic model and Higuchi model for both of the formulations. These values are nearer to 1; so, this model was considered to be a good fit [77]. As reported in [78], based on the composition and morphology of nanoparticles, a different mechanism of drug release could be expected, that is, drug diffusion, matrix degradation, or swelling. Because the in vitro release studies of MRN from SLNs were performed in the absence of enzymes, the degradation of the lipidic matrix cannot be considered as a mechanism for MRN release nor swelling due to the lipophilic nature of the matrix. It is conceivable that drug diffusion is the predominant mechanism that occurs in vitro for MRN release from SLNs. On the other hand, the release profiles reported in Figure 7 demonstrate an SLN structure characterized by the presence of a shell enriched with MRN. Therefore, first-order diffusion was expected, depending on the residue drug within the system, the diffusion distance inside the nanosystems, and the reversible drug–lipid association [72].

According to the Korsmeyer–Peppas model, data obtained from in vitro drug release studies were plotted as log cumulative percentages of MRN released versus log time. The value of transport exponent (*n*) was used to predict the drug release mechanism. We obtained *n* in the range of 0.45 < *n* < 0.89 for both of the systems, evidencing that non-Fickian diffusion occurs as the predominant release mechanism for MRN [76].

### 3.3. Ex Vivo Permeation Experiments

To evaluate the ability of SLNs to improve the permeation of MRN through the biomembranes, an ex vivo permeation study was performed on excised bovine nasal mucosa mounted on Franz’s cells. Studies are present in the literature that demonstrate the potentiality of this method to explore the mechanistic aspects of nasal transport and the metabolism of drugs, representing a valid ex vivo model for drug screening before their application in in vivo studies [79,80,81].

**Figure 8:**
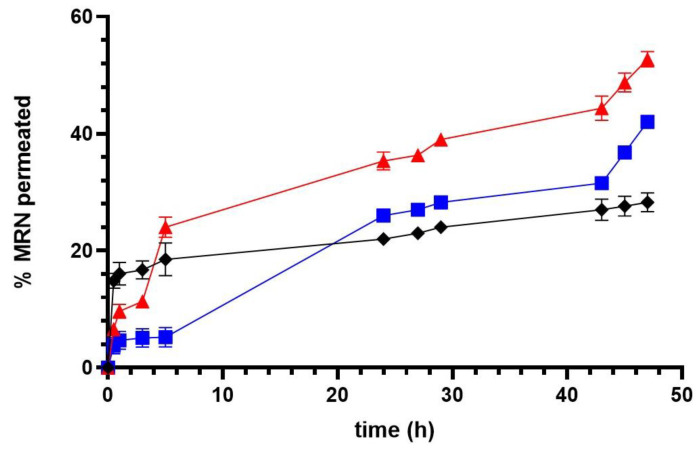
Permeation profiles across the excised bovine nasal mucosa of free MRN (black line), MRN-SLNs-PHO_10_ (red line), and MRN-SLNs-COM_10_ (blue line). Results are expressed as mean values of three different experiments from three different batches ± standard deviation. If bars are not visible, they are within the symbol. All data related to MRN-SLNs are statistically significant with respect to free MRN data (*p* < 0.001).

In Figure 8, we showed the permeation profiles obtained for free MRN and MRN encapsulated into SLNs over the experimental time (48 h). All of the samples were prepared in PBS (pH 7.4); so, not only were the formulations in suspension, but the free MRN was too, due to its low solubility in this solvent (55 μg/mL in PBS pH 7.4 [26]). For this reason, the ability of free MRN to cross mucosa is related to its physical–chemical properties, i.e., its intrinsic solubility, dissolution rate, and lipophilicity [82]. The biopharmaceutical classification system in fact described the aqueous solubility and membrane permeability of a drug as the main factors that limit drug absorption via the viable membrane [83]. As demonstrated by Li et al. [84], at pH 7.04, MRN has a Log D lower than 1 and a percentage of unionized form of about 1.21%, suggesting that the poor solubility and low permeability of MRN are the limiting steps to its absorption through the biomembranes [84].

**Figure 9:**
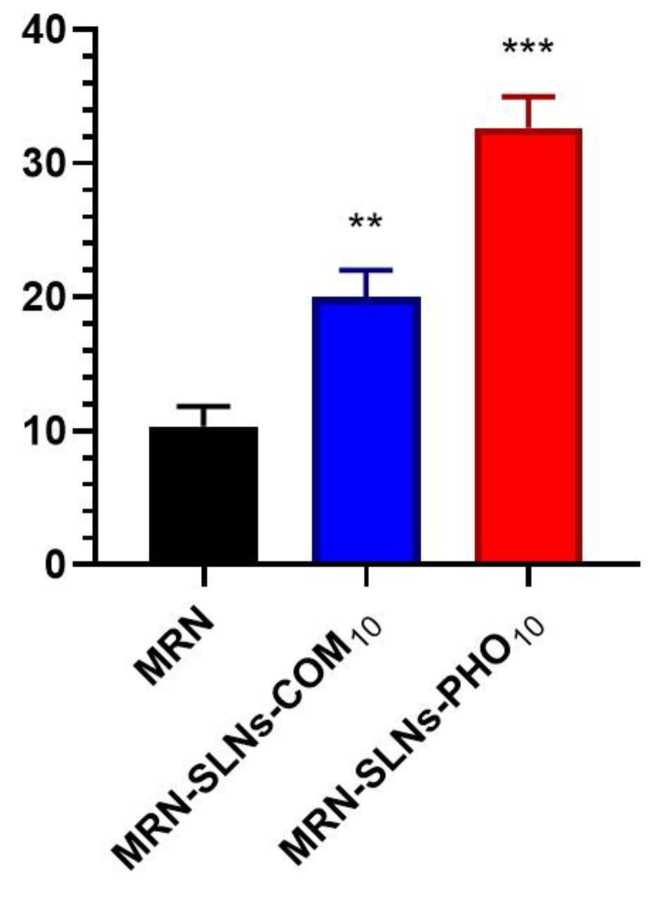
Amounts of free MRN and MRN-loaded SLNs accumulated into the excised bovine nasal mucosa at the end of the permeation experiment. Results are expressed as mean values of three different experiments from three different batches ± standard deviation. If bars are not visible, they are within the symbol. *** *p* < 0.001 and ** *p* = 0.003 versus free MRN.

These observations permitted us to exploit the permeation profile obtained for the free drug in our experiment. The permeation observed for free MRN in the first hour of the experiment (about 15%, *w*/*w*) could be related to the low amount of MRN in the solution which, due to its lipophilicity, was available to cross the mucosa. After that, the permeation proceeded very slowly and could reflect the low rate of MRN dissolution. To confirm this hypothesis, the accumulated MRN in the treated mucosa was quantified at the end of the experiment. A very low value was obtained of about 10% (*w*/*w*) of the assayed dose (Figure 9), demonstrating the scarce availability of the free drug to cross the biomembranes. 

Different results were obtained for both of the formulations. They showed a progressive increase in the permeated MRN, and within 48 h, about 40% (*w*/*w*) and 50% (*w*/*w*) of MRN was detected in the receptor compartment for MRN-SLNs-COM_10_ and MRN-SLNs-PHO_10_, respectively. This result could be related to the amount of the free drug released by the formulations and immediately available for permeation. In fact, higher in vitro drug release for MRN-SLNs-PHO_10_ with respect to MRN-SLNs-COM_10_ corresponded to a higher percentage of permeated MRN. Due to the lipidic composition of the realized delivery systems, an intimate contact with the mucosa could not be excluded, also improved by the presence of surfactant on the particle surfaces [85], resulting in increased permeation of encapsulated MRN with respect to the free drug. Furthermore, based on the little sizes of our formulations (<200 nm), the internalization of the MRN-SLNs into the mucosa could not be excluded [86]. In this way, MRN-SLNs accumulated within the nasal mucosa could represent a deposit able to produce the prolonged release of the drug. This last hypothesis could be confirmed by the MRN accumulated in the mucosa after 48 h treatment with the two formulations; it was higher with respect to that observed for the free drug (about 20% and 35%, *w*/*w* for MRN-SLNs-COM_10_ and MRN-SLNs-PHO_10_, respectively) (Figure 9). On the other hand, various studies [85,86,87] have demonstrated SLNs as being an optimum carrier for increasing the biodistribution of drugs into the brain via nasal administration, due to their ability to protect drugs from chemical and enzymatic degradation [88], to improve permeation through nasal mucosa due to their lipids and surfactant composition, and to promote active or passive targeting due to their small sizes and superficial charge [86].

## 4. Conclusions

In this study, we performed a preformulation study to optimize SLN formulations intended for the nose-to-brain targeting of MRN. We used two different lipids, that is, PHO and COM, obtaining similar results concerning sizes, zeta potential, encapsulation efficiency, and drug loading. The small sizes and spherical morphology of the realized SLNs makes them suitable for nasal administration. Both of the formulations showed sustained drug release and prolonged permeation through the excised bovine nasal mucosa, compared to the pure drug. Furthermore, the MRN-SLNs allowed for a greater accumulation of the drug inside the mucosa with respect to the free MRN, probably due to the internalization of the SLNs into the mucosa. 

Although biological in vitro and in vivo studies are needed, based on our data, we believe that MRN-SLNs are promising systems for the potential treatment of neurodegenerative diseases based on oxidative injury.

## Figures and Tables

**Table 1:** Yield (%) and encapsulation parameters (E.E. (%), D.C. (%)) of MRN-SLNs prepared using COM (MRN-SLNs-COM) and PHO (MRN-SLNs-PHO) as lipids and with different theoretical amounts of MRN.

Theoretical MRN Amount (%, *w*/*w*)	MRN-SLNs-COM			MRN-SLNs-PHO		
	E.E. % ± SD	D.C. % ± SD	Yield % ± SD	E.E. % ± SD	D.C. % ± SD	Yield % ± SD
0	---	---	50.7 ± 0.5	---	---	54.42 ± 2.5
2	84.87 ± 0.1	0.69 ± 0.8	52.5 ± 0.8	87.86 ± 1.2	0.73 ± 2.2	51.55 ± 3.1
5	87.23 ± 1.0	1.79 ± 0.7	52.02 ± 3.9	88.29 ± 0.2	1.8 ± 5.4	52.44 ± 1.6
8	95.58 ± 0.6	3.15 ± 4.5	51.6 ± 5.2	98.12 ± 0.8	3.48 ± 1.3	48.01 ± 0.4
10	99.23 ± 0.2	4.01 ± 4.4	52.52 ± 4.2	99.03 ± 0.3	4.27 ± 2.4	49.17± 1.1

**Table 2:** Hydrodynamic radius, (R_H_), polydispersity index (PDI), and zeta potential (ζ) values of blank SLNs, MRN-SLNs-COM_10_, and MRN-SLNs-PHO_10_ samples.

Samples	RH (nm)	PDI (%)	ζ (mV)
**Blank SLNs-COM**	60.8 ± 8.5	0.19 ± 0.1	-30.3 ± 1.2
**MRN-SLNs-COM_10_**	76.5 ± 5.6	0.257 ± 0.3	-32.0 ± 1.6
**Blank SLNs-PHO**	45.3 ± 12.5	0.15 ± 0.5	-23 ± 2.5
**MRN-SLNs-PHO_10_**	72.95 ± 6.2	0.298 ± 0.05	-22.3 ± 3.3

**Table 3:** Regression coefficient (R^2^), rate constant (K_i_, i = 0 for zero-order, 1 for first order, and H for Higuchi model, respectively), and transport exponent (*n*) of Korsmeyer–Peppas model, as obtained from release data of MRN from SLNs.

	Zero-Order		First-Order		Higuchi		Korsmeyer–Peppas	
	R^2^	K_0_ (d^−1^)	R^2^	K_1_ (d^−1^)	R^2^	K_H_ (d^−1/2^)	R^2^	*n*
**MRN-SLNs-COM_10_**	0.7063 ±0.0023	0.4141	0.9326 ±0.0072	0.013217	0.8989 ±0.0023	7.0212	0.9327 ±0.0042	0.838
**MRN-SLNs-PHO_10_**	0.6577 ±0.0132	0.39894	0.9113 ±0.022	0.014899	0.8742 ±0.0092	6.9102	0.8273 ±0.0021	0.619

## Data Availability

Data are contained within the article.

## Supplementary Materials

The following supporting information can be downloaded at https://www.mdpi.com/article/10.3390/pharmaceutics15061605/s1: Figure S1: MRN chromatogram (100 μg/mL concentration). See Materials and Methods section for the operative conditions. Figure S2: Plot of peak area vs. MRN concentration.
