# Supplementary material for: Solid Lipid Nanoparticles Containing Morin: Preparation, Characterization, and Ex Vivo Permeation Studies
Source: Pharmaceutics. 2023 May 28;15(6):1605. doi: 10.3390/pharmaceutics15061605 (PMC10300871; doi:10.3390/pharmaceutics15061605)
Supplement: Supplementary file 1 [file pharmaceutics-15-01605-s001.zip › pharmaceutics-2398313-supplementary.pdf]

pharmaceutics

Supplementary Materials

Solid lipid nanoparticles containing Morin: preparation, characterization, and *ex vivo* permeation studies.

Federica De Gaetano[1], Consuelo Celesti[2], Giuseppe Paladini[3], Valentina Venuti[4], Maria Chiara Cristiano[5], Donatella Paolino[6], Daniela Iannazzo[2], Vincenza Strano[7], Anna Gueli[3], Silvana Tommasini[1], Cinzia Anna Ventura[1*], Rosanna Stancanelli[1]

[1] Department of Chemical, Biological, Pharmaceutical and Environmental Sciences, University of Messina, V.le Ferdinando Stagno d'Alcontres 31, I-98166 Messina, Italy; fedegaetano@unime.it; stommasini@unime.it; rstancanelli@unime.it
[2] Department of Engineering, University of Messina, Contrada Di Dio, I-98166 Messina, Italy; ccelesti@unime.it
[3] Department of Physics and Astronomy "Ettore Majorana", University of Catania, Via S. Sofia, 64 I-95123 Catania, Italy; anna.gueli@unict.it
[4] Department of Mathematical and Computer Sciences, Physical Sciences and Earth Sciences, University of Messina, V.le Ferdinando Stagno D'Alcontres 31, I-98166 Messina, Italy; vvenuti@unime.it
[5] Department of Medical and Surgical Sciences University of Catanzaro "Magna Graecia", V.le Europa s.n.c., I-88100 Catanzaro, Italy; mchiara.cristiano@unicz.it
[6] Department of Experimental and Clinical Medicine, University of Catanzaro "Magna Graecia", V.le Europa s.n.c., I-88100 Catanzaro, Italy; paolino@unicz.it
[7] CNR-IMM, Catania (University) Unit, Via S. Sofia 64, I-95123 Catania, Italy; vincenzina.strano@ct.infn.it
* Correspondence: caventura@unime.it (C.A.V.)

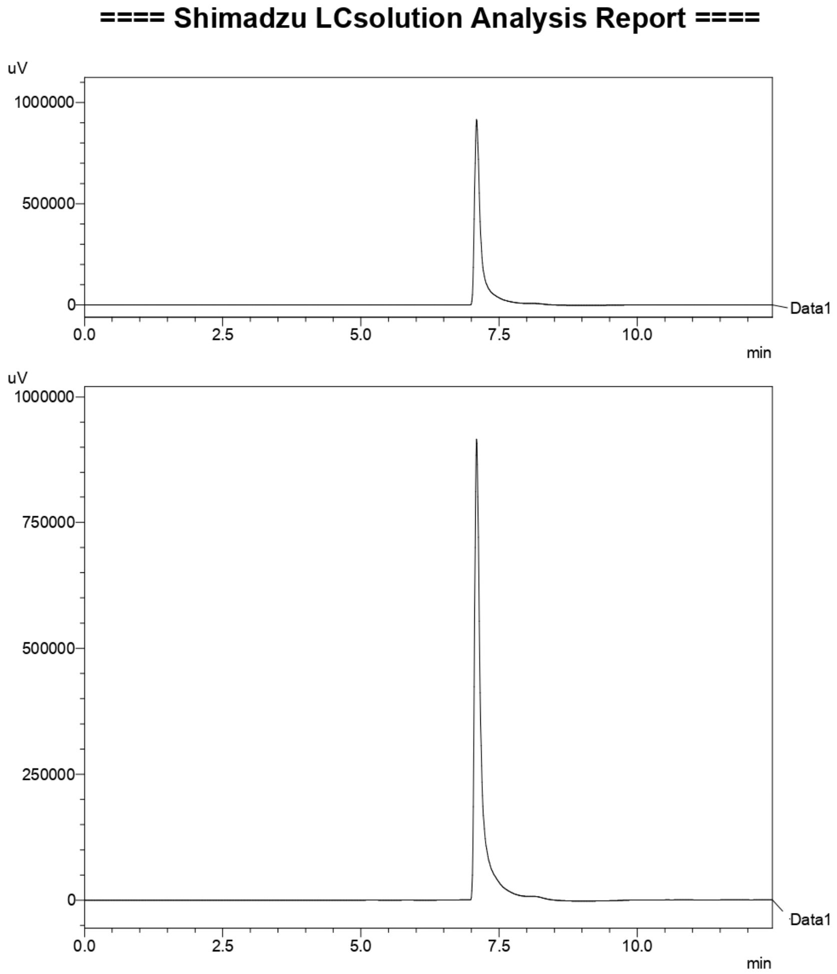

Figure S1. MRN chromatogram (100 µg/mL concentration). See material and methods section for the operative conditions.

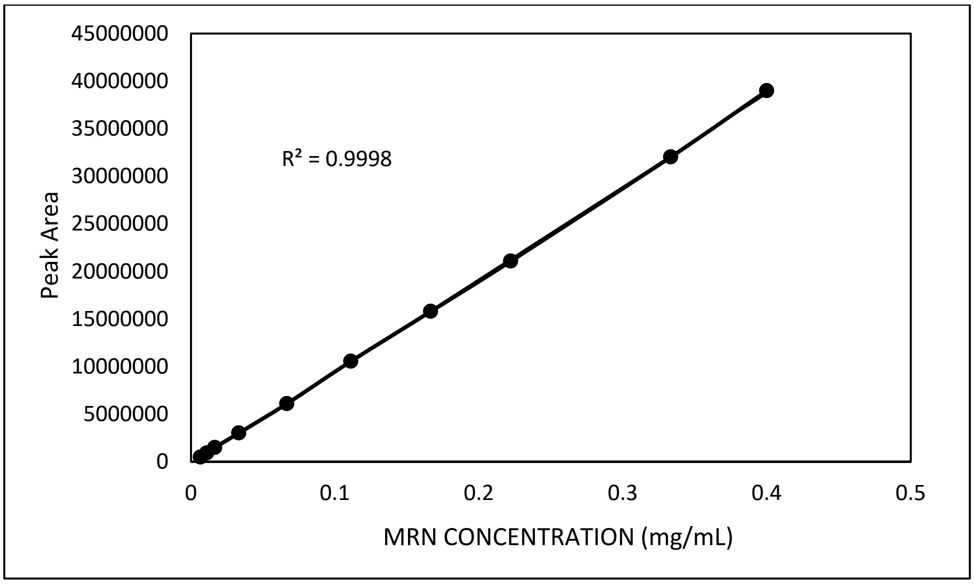

Figure S2. Plot of peak area vs. MRN concentration
